# Predictors of rural hospital closures in the United States: a systematic review and call for AI-driven early warning systems

**DOI:** 10.1186/s12913-025-13847-7

**Published:** 2025-12-13

**Authors:** Kiruthika Balakrishnan, Zhi Li, Hana E. Hinkle, Vicki L. Weidenbacher-Hoper, Cynthia Reynolds, Avery O. Keesee

**Affiliations:** 1 University of Illinois College of Medicine Rockford, National Center for Rural Health Professions, 1601 Parkview Avenue, Rockford, IL 61107 USA; 2 Regional Health Sciences Librarian and Instructor, University of Illinois College of Medicine Rockford, 1601 Parkview Avenue, Rockford, 61107 IL USA

**Keywords:** Rural hospital closures, Financial distress and workforce shortages, Medicaid expansion, Predictive analytics, Early warning systems

## Abstract

**Background:**

Rural hospitals in the U.S. are closing at an alarming rate, threatening healthcare access and economic stability in underserved communities. While numerous studies have explored the causes and impacts of these closures, a predictive framework to proactively identify at-risk hospitals early remains underdeveloped.

**Objective:**

This systematic review examines the financial, workforce, policy-related, economic, and demographic pressures, as well as market dynamics that contribute to rural hospital closures and evaluates the extent to which current research supports the use of AI-driven early warning systems to predict and prevent future closures.

**Methods:**

A systematic literature search was conducted across PubMed, Embase, CINAHL Plus, and Scopus. Studies published between 2013 and 2024 were screened based on predefined inclusion and exclusion criteria. A total of 15 studies were included and analyzed using a narrative synthesis framework following PRISMA guidelines.

**Results:**

The review identified five primary categories contributing to rural hospital closures: financial distress, workforce shortages, unfavorable policy environments, adverse economic and demographic conditions, and market competition. Most studies employed traditional statistical models and used retrospective data to analyze these issues. None of the studies applied ML models to identify these factors. However, there is a significant gap in the availability of real-time tools that can identify contributing factors, predict closures in advance, and guide timely interventions.

**Conclusion:**

Despite the increasing potential of AI, no studies included in this review have applied ML techniques to predict or prevent rural hospital closures. These findings highlight a critical need for developing real-time, AI-driven early warning systems, such as the proposed Rural Health Control Tower (RHCT), to continuously monitor multi-source data, issue dynamic risk alerts, and support proactive decision-making. The predictors and data sources identified in this review offer a foundation for developing such models, which could improve the timeliness of interventions and promote the sustainability of rural healthcare. Future research should prioritize the development of interpretable, equitable, and community-informed AI tools that are accessible to all.

**Clinical trial number:**

Not applicable

**Supplementary Information:**

The online version contains supplementary material available at 10.1186/s12913-025-13847-7.

## Introduction

In 2023, approximately 55.94 million people resided in rural areas of the U.S., making up about 17.9% of the total U.S. population [1]. Rural hospitals in non-metropolitan regions serve as the primary healthcare providers for these communities, offering essential medical services when access to larger healthcare systems is limited [2]. These hospitals vary in size and scope of service, but typically provide primary care, emergency care, maternity care, chronic disease management, and long-term care. However, despite their critical role in healthcare delivery, rural hospitals in the U.S. have experienced an increasing rate of closures over the past two decades. Since 2010, 152 rural hospitals across the U.S. have either closed or changed the services they offered. Among these, 88 hospitals have completely shut down, while 64 have ceased inpatient services but continue to provide limited care options, such as primary care, skilled nursing, or long-term care [3]. This rising trend of hospital closures has far-reaching consequences, particularly affecting healthcare access and the economic stability of rural communities.

Rural hospital closures have significant consequences for healthcare accessibility. One immediate effect is the increased travel distance to medical facilities, which delays emergency response times and limits access to essential healthcare services [4]. Recent geospatial analyses reveal that up to 0.97% of the U.S. population has lost access to a hospital within a 15-minute drive [5], with many rural residents now facing travel times exceeding 30 minutes and distances greater than 5 miles to receive care [6]. The most severe impacts have been observed in the East South Central region, where 0.97% of residents (178,478 individuals) are affected, and in the West South Central region, where 0.54% of residents (197,660 individuals) are affected. This issue is particularly pronounced among rural populations, as some individuals now must travel more than 20 miles to reach the nearest hospital [5].

In addition to emergency care challenges, hospital closures increase the burden on nearby healthcare institutions. As rural hospitals shut down, inpatient admissions and emergency department visits rise at neighboring facilities, straining their operational and financial resources. This issue is particularly pronounced in the South and Appalachia, where most closures have occurred [7]. A study found that hospital closures have led to an 8.7% rise in inpatient mortality for time-sensitive conditions such as sepsis, stroke, and acute myocardial infarction [8]. The impact disproportionately affects Medicaid patients and racial minorities. However, other studies have suggested that hospital closures do not always lead to measurable increases in local hospitalization rates or overall mortality, highlighting the complexity of these outcomes [9].

Despite the increased presence of Federally Qualified Health Centers (FQHCs) within 10 miles of closed hospitals, rural communities still face persistent unmet needs for preventive and therapeutic care [10]. Additionally, hospital closures contribute to long-term physician shortages. Following a hospital closure, the supply of primary care physicians declines by an average of 8.2% annually for at least six years, with significant reductions in general surgeons and surgical specialists as well [11]. These workforce shortages further exacerbate healthcare access challenges in affected communities.

Beyond access to healthcare, the closure of rural hospitals has significant economic repercussions. The closure of a hospital can accelerate population decline, hinder economic growth, and elevate poverty levels [12]. Research indicates that when a rural county loses its only hospital, the area faces a long-term decline in per capita income of up to 4%, along with a 1.6%-point increase in unemployment rates. These economic downturns have a ripple effect on local businesses and workforce retention, leading to broader financial instability [13]. Similarly, Malone et al. found that rural hospital closures resulted in an average 1.4% decrease in the labor force size and a 1.1% decline in population size in affected counties [14]. Additionally, Vogler (2020) estimated that hospital closures led to a 1.8% drop in non-hospital employment, further disrupting businesses that rely on hospital-driven economic activity [15].

However, some studies report mixed economic effects. Using a quasi-experimental approach, Ona et al. found no statistically significant income differences between counties that experienced hospital closures and those that did not [16]. Similarly, Pearson et al. analyzed 24 rural counties in Texas and found no significant short- or long-term negative impacts on local economies following hospital closures [17]. These findings suggest that economic impacts vary based on regional factors, hospital size, and the availability of alternative healthcare facilities.

In many instances, hospitals are among the largest employers in rural communities. Their closures lead to job losses that extend beyond healthcare workers, affecting the local economy as a whole. Moreover, the loss of healthcare services makes these regions less appealing to new businesses and residents, further undermining economic stability.

Numerous studies have examined the impact of rural hospital closures on healthcare access and economic conditions [12], yet the underlying causes of these closures remain complex. Existing research highlights financial instability, policy-related challenges, workforce shortage, and demographic shifts as primary contributors to the rural hospital closures [17, 18]. While many studies focus on these factors, only a few attempt to predict future distress in rural facilities. One notable model predicts financial distress in rural hospitals using publicly available financial, organizational, and market data, achieving a strong predictive performance [19]. Similarly, Holmes et al. developed and validated a Financial Distress Index (FDI) that utilizes both hospital and community characteristics to predict rural hospital financial distress and the likelihood of closure within two years [20]. Their model shows improved specificity and predictive power for rural hospital closures; however, it primarily relies on retrospective data and traditional statistical analyses, lacking real-time predictive capabilities (Table 1 compares existing models and their limitations).

**Table 1:** Existing models for predicting rural hospital financial distress and their limitations

Authors & Year	Variables Used for Analysis	Model Used	Limitations
Malone et al. [19] & 2024	Financial performance, government reimbursement, organizational traits, and market characteristics	Probit regression model; AUC = 0.87	Not integrated into real-time automated decision support, all of which limit its utility for proactive early warning.
Holmes et al. [20] & 2017	Financial and community data	Logistic regression model (Financial Distress Index); c-statistic = 0.74	Not designed for dynamic, adaptive learning or integration into continuous monitoring, limiting its role as an early warning tool.

Beyond financial instability, rural hospital closures often arise from the cumulative interaction of economic, operational, workforce, and community-level stressors that intensify over time. Financial distress typically begins well before closure, reflected in declining margins, shrinking patient volumes, and workforce shortages that gradually undermine sustainability. Studies, such as those by Malone et al., demonstrate that economic pressures, particularly narrowing profit margins and regional market consolidation, significantly contribute to the risk of closure [14]. Likewise, Holmes et al. found that smaller hospitals with limited government reimbursement and weaker liquidity are at a higher risk of financial distress [20]. Although one recent international study demonstrated the feasibility of using machine learning (ML) to predict the closure of smaller healthcare facilities using administrative health data, such efforts remain scarce for rural hospitals in the U.S. [21]. These findings collectively suggest that early signs of instability are observable well before closure, yet traditional analytical methods often fail to capture these patterns in real time. This limitation highlights the need for analytic frameworks that can continuously process large, multidimensional data inputs to detect emerging risk trajectories before they escalate to crisis-level conditions.

Given this pattern of delayed recognition and reactive intervention, there is an urgent need for an AI/ML-enabled early warning system that integrates financial, workforce, and community-level data to anticipate distress before it becomes irreversible. Empirical research by Rhoades et al. demonstrates that sociodemographic factors, including unemployment and insurance coverage, have a significant impact on rural hospital survival [22]. In contrast, Kaufman et al. identified liquidity, debt burden, and payer mix as quantifiable predictors of closure risk [23]. We hypothesize that these tools could analyze and identify predictors of rural hospital closures, thereby providing early warning signs to facilitate targeted interventions that prevent closures. Despite its proven effectiveness in other areas, AI remains underutilized in addressing this issue [21]. AI models are especially well-suited for this task because they can uncover non-linear relationships and complex interactions between various data sources that traditional statistical models might overlook [24].

AI-driven early warning systems have shown significant potential in the corporate finance, insurance, and banking sectors, as illustrated in Table 2, and provide valuable insights for healthcare applications, such as predicting the closure of rural hospitals. In corporate finance, multi-stage financial distress models that use random forest algorithms have effectively detected transitions from an active to an insolvent status and from an insolvent to a bankruptcy status. These models outperform traditional single-stage bankruptcy predictions by identifying risk signals earlier and making them more actionable [25]. In the insurance sector, ML models, including XGBoost, random forests, and neural networks, have been applied to extensive datasets of financial ratios to forecast capital requirements up to a year in advance, enabling regulators to prioritize interventions and mitigate solvency risks [26]. Similarly, in systemic banking risk prediction, expert voting frameworks based on random forests have shown improved generalization compared to conventional panel logit models. This success is due to their ability to integrate diverse macroeconomic, financial, and structural indicators while accommodating nonstationary data and outliers [27]. Furthermore, ML models have been successfully employed to provide early warnings for clinical deterioration [29] and disease outbreaks [30]. AI-driven health surveillance systems, such as China’s Intelligent Infectious Disease Active Surveillance system, leverage multi-source data and ML models to predict transmission risks and automate early warnings [28]. These findings highlight the potential to adapt multidimensional AI frameworks to identify and mitigate risks associated with rural hospital closures proactively.

**Table 2:** Summary of representative AI/ML-based early warning systems

Study & Year	Domain	AI/ML Model(s)	Performance	Real-World Potential
Tanaka et al., & 2025 [25]	Corporate insolvency	Random Forest (top performer), compared with XGBoost and Logistic Regression.	AUC ≈ 0.93	Enables early detection of financial distress in companies
Kocer et al. & 2025 [26]	Insurance solvency	XGBoost, Random Forest, Gradient Boosting, Neural Network	Accuracy up to 99%	Can serve as a regulatory monitoring tool for insurers
Wang et al., & 2021 [27]	Systemic banking crisis	Random Forest experts voting EWS, Decision Trees, Boosting	AUC > 80%	Global EWS adaptable to heterogeneous economies
Kang et al., & 2024 [28]	Infectious disease outbreak prevention (China’s Intelligent Infectious Disease Active Surveillance & Early Warning System)	Ensemble of models in the model market, including ARIMA, Support Vector Machine, XGBoost, LSTM, Random Forest, plus NLP-based data integration	Forecast horizon: 7–30 days; triggered 391 early warnings in ~ 6 months	Real-time, multi-source, automated early detection and risk assessment enable targeted interventions for outbreak containment

Applying similar techniques to develop predictive models of rural hospital closures could help stakeholders intervene early and address the underlying factors driving hospital closures [21]. Although some studies have explored financial metrics to predict closures, a comprehensive approach integrating financial, operational, policy, and demographic factors through AI-driven models remains largely unexplored. However, challenges such as data availability, quality, and granularity in rural areas continue to impede the development of effective predictive models. Additionally, concerns exist about potential algorithmic bias and the need for interpretable models that policymakers and hospital administrators can trust. Addressing these challenges is crucial for developing effective early warning systems.

By integrating insights from prior studies that did not implement AI-based early warning systems to prevent rural hospital closures, this systematic review takes a critical first step toward identifying the key predictors needed to develop such models. By exploring the potential of predictive analytics to generate early warning signals, this study seeks to bridge the gap between traditional risk factors and emerging technological solutions. This review aims to identify the financial, operational, policy-related, and community-level factors contributing to rural hospital closures in the U.S. and assess the extent to which AI and ML techniques have been applied in this context. Through a systematic review of the literature, we aim to identify these contributing factors and relevant data sources while highlighting areas for future research. Our ultimate goal is to provide actionable insights that inform the development of AI-driven interventions aimed at preventing closures and enhancing healthcare sustainability in vulnerable rural communities.

## Methodology

A systematic literature review was conducted to answer the following question: *What financial, operational, policy-related, and community-level factors contribute to the closure of rural hospitals in the United States (U.S.)?* In addition to identifying these contributing factors, we examined whether the studies in our review used ML, AI, or predictive modeling to support early warning systems. This study did not require Institutional Review Board (IRB) approval due to its design.

### Search strategy

Searches were conducted using PubMed, Embase, CINAHL Plus (EBSCOhost), and Scopus. Our search focused on keywords and database-specific subject headings related to rural hospitals and hospital closures. No restrictions were imposed concerning the date or language. Librarians who specialize in literature reviews developed the search strategies. The complete and reproducible search strategies for all included databases are in the Appendix/Supplementary Materials. The search was conducted across all databases on January 15, 2025.

### Inclusion, exclusion criteria, and screening

All articles obtained from the search were imported into Covidence, a widely used software for screening literature reviews [31]. Covidence effectively identified and removed duplicate citations from multiple databases. Two reviewers independently screened the titles and abstracts to identify studies focused on the reasons behind rural hospital closures. During this process, studies published before 2013 were excluded, and the focus was narrowed to English-language publications from the U.S. to ensure relevance to recent rural hospital closures. Additionally, the reviewers included only studies that applied a clear definition of “rural” to identify their study population or sample. Accepted definitions included the Office of Management and Budget (OMB) designation of non-metropolitan counties, Rural-Urban Commuting Area (RUCA) codes of 4 or greater, Rural-Urban Continuum Codes (RUCC), and Critical Access Hospital (CAH) status as defined by the Centers for Medicare and Medicaid Services (CMS), along with other commonly accepted rural classification systems. Editorials, letters, review articles, and commentaries were also excluded to differentiate original research from non-original contributions. Any disagreements or uncertainties regarding the eligibility of studies for full-text review were resolved through discussion between the reviewers.

Each selected manuscript on rural hospital closures underwent a detailed evaluation, considering key aspects such as study design (including quantitative, qualitative, and mixed-method approaches) and relevance to the financial, operational, policy-related, and community-level factors influencing closures. Studies were assessed for methodological rigor, scope, and alignment with the research objectives to develop a comprehensive understanding of the causes of rural hospital closures in the U.S. Finally, the two reviewers independently reviewed the selected manuscripts and extracted relevant quantitative and qualitative findings on the reasons for rural hospital closures, which contributed to a narrative synthesis of the existing literature.

We developed a standardized data extraction form to chart the relevant characteristics of each systematically included study. The extracted data included the author names, year of publication, study title, stated objective, and methodological design. We recorded the type and source of data used, including national hospital datasets, state-level administrative records, and financial databases. The synthesis also included a detailed review of the dependent and independent variables analyzed, including hospital closure status, operating margins, patient demographics, and policy exposure.

### Data synthesis

**Fig. 1:**
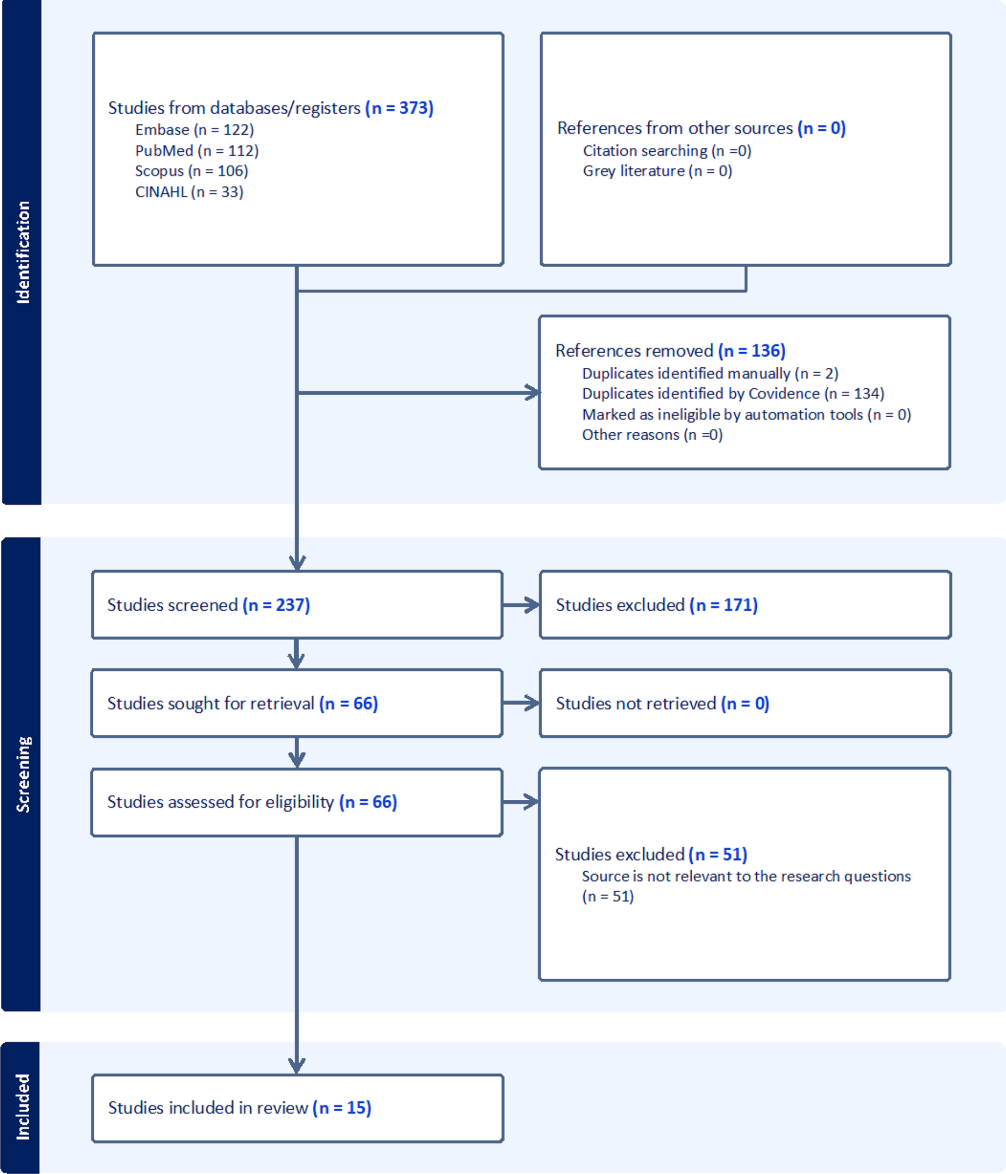
PRISMA flowchart diagram for the proposed review

The Preferred Reporting Items for Systematic Reviews and Meta-Analyses (PRISMA) guidelines [32] were followed to ensure transparency and rigor throughout the review process (Fig. 1). The results of the included studies were synthesized and organized into six major thematic categories aligned with the objectives of this review: (1) a summary of the objectives and descriptions of the included studies; (2) sources of data and information utilized; (3) key factors analyzed to identify the causes of rural hospital closures; (4) methodological approaches employed across the studies; (5) specific factors contributing to rural hospital closures as identified in the literature; and (6) limitations reported in the included studies. Each article was evaluated based on the methodological rigor and reliability of the reported findings regarding rural hospital closures.Fig. 1PRISMA flowchart diagram for the proposed review

## Results

A total of 373 studies were imported from various databases for screening: Embase (*n* = 122), PubMed (*n* = 112), Scopus (*n* = 106), and CINAHL Plus (*n* = 33). After removing duplicates, 237 studies remained. Following a review of titles and abstracts, 66 articles were identified as potentially relevant to rural hospital closures. However, 51 were excluded for the following reasons: lack of empirical data or relevant quantitative/qualitative analysis, focus on non-U.S. settings, examination of hospital financial performance without closure outcomes, or analysis of the impact of rural hospital closures rather than their predictors. After thoroughly reviewing the full texts, 15 manuscripts were ultimately included in the final analysis (see Fig. 1 for PRISMA flow diagram). The detailed metadata for the narrative synthesis of the 15 studies included in this review are provided in Supplementary Table S1.

### Summary of the objectives and descriptions of the included studies

This analysis examines 15 studies that investigate the various factors contributing to the closure of rural hospitals in the U.S. The key challenges identified include financial distress, workforce shortages, policy influences, economic and demographic influences, and market dynamics.

### Financial distress

Financial distress remains the primary driver of rural hospital closures. Several studies have investigated financial sustainability and developed predictive models to assess financial risk. One study analyzed the financial performance of 1004 rural hospitals between 2011 and 2017, assessing differences in profitability by ownership and designation type, and evaluating the impact of Medicaid expansion on hospital sustainability [33]. Another study introduced and validated a Financial Distress Index (FDI) to predict closure risks among 2466 rural hospitals, categorizing them based on indicators such as unprofitability, equity decline, and insolvency [20]. Research examining Medicare Advantage penetration in rural areas assessed how increased enrollment in these plans affected hospital financial stability and closure risk between 2008 and 2019, using financial distress models to analyze hospital market characteristics [34]. Additionally, a study of rural hospitals’ survival strategies from 2010 to 2018 explored how financially struggling hospitals either closed, merged, or remained operational, emphasizing the role of financial health and market competition [35].

A qualitative study further examined financial distress by analyzing the relationship between community characteristics and hospital closures from 2005 to 2015. This study compared the sociodemographic and market characteristics of closed hospitals with those of operational hospitals, focusing on factors such as population density, market share, unemployment rates, and racial/ethnic composition [36]. These studies provide a comprehensive understanding of rural hospitals’ financial challenges and the strategies they use to respond to financial instability and market pressures.

### Workforce shortages

Workforce shortages significantly impact rural healthcare access and contribute to hospital closures. One study identified hospital- and county-level factors affecting the closure of obstetric units in rural areas, using a mixed-methods approach combining multivariate logistic regression with qualitative interviews with administrators from 306 rural hospitals across nine states [37]. Another study examined the effects of labor and delivery unit closures in rural Georgia from 2012 to 2016, incorporating quantitative hospital and regional data with qualitative assessments from newspaper reports. Findings highlighted the disproportionate impact of closures on Black and low-income women [38]. Additionally, research on the urbanization of American surgery projected significant shortages in general surgery, orthopedic surgery, and obstetrics/gynecology for rural hospitals. By analyzing population trends, surgeon certification data, and hospital distribution statistics, the study estimated future recruitment needs and financial implications for rural healthcare facilities [39].

Further qualitative studies investigated the impact of workforce shortages from the perspective of healthcare professionals. One study explored nurses’ experiences working in rural hospitals that closed between 2014 and 2020. Using a retrospective qualitative approach, researchers conducted semi-structured interviews with nurses from two closed hospitals, identifying themes related to pre-closure conditions, closure dynamics, and the impact on nursing staff and communities [40]. These studies collectively highlight the crucial role of workforce shortages in shaping rural hospitals’ ability to maintain essential services.

### Policy influences

Few studies have explored the impact of healthcare policies on rural hospital closures. One study examined the relationship between Medicaid expansion and rural hospital viability, testing the hypothesis that expanding Medicaid eligibility reduces uncompensated care costs and strengthens hospital financial stability. Using financial data from 2008 to 2016, the study compared closure rates between states that expanded Medicaid and those that did not [41]. Additionally, a study examined the effects of telehealth policy adoption on rural hospital financial outcomes, assessing whether the expansion of remote healthcare services affected hospital revenue distribution, credit ratings, and closure risks. Using a quasi-experimental design based on staggered adoptions of telehealth parity laws, the study measured changes in patient volume, financial performance, and access to capital [42]. These studies highlight the significant role of healthcare policies, such as Medicaid expansion and telehealth adoption, in influencing rural hospital financial stability and closure risks.

### Economic and demographic influences

Economic and demographic influences also play a crucial role in rural hospital closures. One study examined whether community sociodemographic factors, such as unemployment and uninsured rates, affected the survival of financially distressed rural hospitals from 2010 to 2019. It analyzed a sample of at-risk hospitals, employing statistical models to identify key predictors of hospital survival [22]. Another study assessed the economic consequences of rural hospital closures on local communities, using a difference-in-differences analysis of 2094 rural counties to examine changes in unemployment rates, labor force participation, per capita income, and healthcare employment before and after hospital closures [43]. These studies underscore the significant impact of economic and demographic factors on rural hospital survival and the broader community.

### Market competition

Market competition is another factor influencing rural hospital closures. One study examined the rate of rural hospital closures from 2010 to 2014, identifying financial and market characteristics that differentiated hospitals that closed from those that remained operational. Statistical tests and regression analysis examined the relationship between financial distress, market factors, and hospital closures [23]. Another study evaluated whether rural hospitals affiliated with larger health systems had lower closure risk between 2007 and 2019, using time-dependent survival models to analyze hospital characteristics, market conditions, and utilization patterns [44]. These studies collectively highlight the role of market competition in shaping rural hospital sustainability, emphasizing how hospital affiliations, local economic conditions, and healthcare consolidation trends influence hospital closures.

### Sources of data and information

The studies included in this review utilized diverse data sources, including hospital cost reports, national health databases, economic statistics, and qualitative interviews, to analyze rural hospital closures. Several studies primarily relied on the Centers for Medicare and Medicaid Services (CMS) hospital cost reports for financial data. For example, Bai et al. assessed trends in hospital financial viability using CMS hospital cost reports and Small Area Health Insurance Estimates from the U.S. Census Bureau [33]. Similarly, Holmes et al. and Kaufman et al. used CMS Medicare Cost Reports, Provider of Services files, Hospital Service Area files, and Nielsen-Claritas population data to analyze financial distress and market risks contributing to hospital closures [20, 23]. While CMS cost reports provide standardized financial data, they may not fully capture operational challenges faced by small rural hospitals that serve a high proportion of uninsured patients or those outside Medicare programs.

Beyond financial data, economic databases were widely used to assess the broader implications of hospital closures. The Sheps Center for Health Services Research (SCHSR) compiles information on hospital closures and financial distress. This SCHSR dataset was used with the CMS Hospital Cost Report Information System (HCRIS), the American Hospital Directory, and the National Bureau of Economic Research to examine hospital closures, mergers, and financial performance [35]. Additionally, SCHSR data, combined with economic data from the Bureau of Labor Statistics (BLS), the Bureau of Economic Analysis (BEA), the U.S. Federal Reserve, and the U.S. Census Bureau, were utilized to assess the economic impact of hospital closures on local communities [43].

In addition to economic factors, healthcare utilization, and patient outcome data were analyzed to evaluate the causes of hospital closures. Several studies used national health databases, such as the Healthcare Cost and Utilization Project (HCUP) State Inpatient Databases (SID), which provided essential financial and utilization metrics for studying Medicaid expansion and hospital survival [22, 41]. Additionally, CMS HCRIS and the American Community Survey (ACS) were used to examine hospital financial trends and rural healthcare access [34].

Several studies looked at data on the hospital workforce and service availability to investigate the effects of workforce shortages on rural hospital closures. The American Hospital Association (AHA) Annual Survey, the American Medical Association (AMA) Master File, and the American Board of Medical Specialties certifications evaluated hospital characteristics, surgeon supply, and urban-rural disparities in surgical workforce demand [39]. Furthermore, the impact of obstetric unit closures was examined using a combination of HCUP SID, the Area Resource File (ARF), and telephone interviews with hospital administrators across nine states [37]. Labor and delivery unit (LDU) closures in rural Georgia were analyzed using data from the Georgia Department of Public Health, the U.S. Census Bureau, and Emory’s MCH Linked Vital Records Data Repository, along with qualitative sources such as newspaper reports [38].

Financial and policy-related datasets were also widely utilized. The Kaiser Family Foundation (KFF) Medicaid expansion database provided insights on state-level Medicaid expansions and their relationship with hospital closures [33]. The Mergent Municipal Fixed Income Database and the Municipal Securities Rulemaking Board (MSRB) analyzed municipal bond data and the financial implications of telehealth policy changes on rural hospitals [42].

Studies examining hospital market competition and mergers used data from Irving Levin Associates (mergers and acquisitions), HCUP Hospital Market Structure Files, and the RAND Corporation state statistics database to explore the role of market forces in rural hospital closures [43, 44]. Finally, qualitative studies relied on semi-structured interviews and community-based data collection. For instance, Smith et al. captured nurses’ perspectives on hospital closures through interviews with healthcare professionals from hospitals that closed in Texas between 2014 and 2015 [40]. Other studies verified hospital closure statuses and rural classifications using direct contact with hospital representatives, websites, and newspaper database searches, and Rural-Urban Commuting Area (RUCA) codes [36].

The data and information indicate that financial distress, workforce shortages, policy influences, economic and demographic factors, and market dynamics all contribute to hospital closures in rural areas. Reviewed studies used cross-sectional and longitudinal approaches, though most focused on static risk factors rather than dynamic time-dependent processes. Addressing these challenges requires a comprehensive policy and economic intervention to sustain rural healthcare systems.

### Key factors analyzed to identify the causes of rural hospital closures

Several studies included in this review investigated various factors related to hospitals, finances, markets, and policies to understand the reasons behind hospital closures and financial distress, particularly in rural areas. Most papers included hospital-level and regional economic indicators, while some focused on financial distress or healthcare accessibility issues. A few studies examined the impact of hospital closures on the labor market, focusing on changes in employment and per capita income.

Common hospital characteristics included ownership type (public, private, non-profit, or for-profit), system affiliation, bed count, occupancy rate, and accreditation status. Financial indicators were extensively analyzed, with studies often considering operating margin, total margin, debt service coverage, reinvestment levels, and payer mix (including Medicaid, Medicare, private insurance, and self-pay). Additionally, some studies examined telehealth adoption, particularly in states with parity laws, as a potential factor supporting hospital sustainability.

Market characteristics varied across studies but often included competition intensity (typically measured using the Herfindahl-Hirschman Index), median household income, unemployment rates, and demographic factors such as age distribution, racial composition, and insurance coverage rates. Geographic factors were frequently examined, including distance to the nearest hospital, access to obstetric services, and availability of medical professionals such as OB-GYNs, nurse-midwives, and family physicians.

Economic conditions were another critical consideration, with studies incorporating per capita income, participation in disability programs, subprime credit scores, and bankruptcy filings. Several papers also analyzed the impact of Medicaid expansion status on hospital closures. Furthermore, one study used an exclusively qualitative approach to describe the contributors to rural hospital closures, the processes involved, and the outcomes from the perspective of licensed rural nurses during a hospital’s closure [40]. Additionally, some studies utilized newspaper reports to document hospital closures, their contributing factors, and the local community’s responses, providing qualitative insights into the broader implications of these shutdowns. Despite differences in study designs, researchers consistently analyzed financial vulnerability, market competition, and regional economic conditions to identify the reasons for rural hospital closures.

### Methodological approaches employed in the included studies

The included studies utilized various quantitative and qualitative methodologies to examine the key factors related to rural hospital closures. Longitudinal and regression-based approaches were commonly used to assess financial viability and predict distress. For instance, longitudinal analyses combined with descriptive and regression techniques were used to evaluate trends in financial sustainability [33]. Logistic regression models incorporating financial, organizational, and market characteristics were developed to predict financial distress and closure risks, including validating an FDI to monitor hospital stability [39].

Multinomial logit models and difference-in-differences (DiD) analyses were widely employed to assess the effects of financial distress, market competition, and policy changes. One study used multinomial logit models to evaluate within-market and out-of-market mergers among rural hospitals [35]. At the same time, another applied a DiD approach to analyze economic shifts following hospital closures [43]. Additionally, DiD was used to investigate the financial effects of telehealth adoption and Medicaid expansion under the Affordable Care Act (ACA) on rural hospital sustainability [41, 42].

Retrospective cohort studies and survival models were also used to examine hospital closures. A study utilizing fixed-effects regression and Cox proportional hazards models explored the relationship between Medicare Advantage penetration and rural hospital financial distress [34]. Another study applied a time-dependent survival model to compare closure risks between independent and multihospital-affiliated rural hospitals [44]. Community sociodemographic factors influencing hospital survival were analyzed using Wilcoxon rank-sum tests and multilevel Weibull proportional-hazards regression [22].

Quantitative analyzes projecting future surgeon availability were used in several studies focused on healthcare workforce shortages and closures. One study quantitatively analyzed and projected future surgeon availability based on population and workforce trends [39]. A mixed-methods study integrating multivariate logistic regression and qualitative analysis examined factors contributing to rural obstetric unit closures and their impact on prenatal care access [37]. Similarly, closures of labor and delivery units (LDUs) in rural Georgia were analyzed using quantitative and qualitative methods, highlighting disparities affecting Black women [38].

Comparative statistical analyses were applied to assess financial and market-related risks associated with rural hospital closures. Pearson’s chi-square and Wilcoxon rank-sum tests were used to evaluate financial and market characteristics influencing hospital closure rates, with logistic regression used to predict financial distress [23]. Multilevel logistic regression compared closed and financially distressed but operational hospitals, emphasizing the role of community characteristics in hospital sustainability [36]. Finally, qualitative research contributed insights into the social and professional impacts of rural hospital closures, including a cross-sectional, retrospective study documenting rural nurses’ experiences and challenges during hospital closures [40]. The studies reviewed used various quantitative and qualitative methods to investigate the reasons behind rural hospital closures. Researchers commonly employed longitudinal and regression-based techniques, such as logistic regression, multinomial logit models, difference-in-differences analyses, and survival models, to predict financial distress and evaluate the effects of policy changes and economic shifts.

### Factors contributing to rural hospital closures identified from the included studies

The included studies identified various factors contributing to rural hospital closures and categorized them into five major categories, as summarized in Table 3. Overall, rural hospital closures are primarily driven by financial distress, declining patient volumes, and adverse economic conditions. Factors contributing to financial instability include low profitability, narrowing profit margins, a high dependency on Medicare and Medicaid reimbursements, and lower Medicare payments than private insurers. Economic downturns, characterized by rising unemployment and declining incomes, further exacerbate this financial distress. One study found that adverse economic conditions are a precursor to hospital closures [43].

**Table 3:** Summary of factors contributing to rural hospital closures

Category	Predictor Variables/Reasons for Closure
Financial Distress	Financial instability and unprofitability, declining operating margins, High debt and poor liquidity, Negative equity and insolvency, Low occupancy rates, Heavy dependence on Medicare/Medicaid reimbursement, non-CAH designation, Small hospital size, For-profit ownership, Delayed government reimbursements, Budget cuts, Financial losses from obstetric or surgical units, Credit-rating downgrades, lack of capital investment
Workforce Shortages	Shortage of physicians, nurses, and specialists, Difficulty recruiting or retaining obstetricians, surgeons, and anesthesiologists, Loss of specialty services, Provider burnout and turnover, Low birth or surgical volumes, Continued urbanization of surgical services
Policy Influences	Lack of Medicaid expansion, Low Medicaid reimbursement rates, Changes in state/federal payment policies, Telehealth parity laws redistributing revenue, Policy shifts causing uncertainty in funding streams
Economic and Demographic Influences	High community unemployment, declining per-capita income, Out-migration/population loss, Aging population, High uninsured or self-pay patient rates, High poverty levels, Adverse local economic conditions, Racial and ethnic disparities, Low market demand and small catchment populations, lack of community support for hospital sustainability
Market Competition	High market competition and low market share, Proximity to other hospitals, Affiliation with multi-hospital systems or mergers, Service consolidation by parent systems, Telehealth expansion drawing patients to urban centers, Loss of obstetric/surgical service lines, administrative mismanagement, Limited community support

Structural changes in healthcare, such as the shift of revenue from rural to urban hospitals facilitated by the expansion of telehealth, have reduced profit margins and credit ratings for rural hospitals. Service line closures, particularly in obstetric and surgical care, have been linked to low birth volumes, high rates of uninsured or self-pay patients, inadequate Medicaid reimbursements, and shortages of healthcare providers. The difficulty in recruiting surgeons and anesthesiologists due to financial constraints has further compromised hospital viability.

Additional factors that increase the risk of closure include market competition, small population sizes, and low reimbursement rates, especially for hospitals operating as for-profit entities or lacking Critical Access Hospital (CAH) designation. Affiliation with multi-hospital systems, proprietary ownership, and reduced market share also contribute to financial distress and hospital closures. Moreover, high rates of uninsured patients, the absence of Medicaid expansion, and increased uncompensated care burdens heighten financial instability.

Policy changes, lack of community support, administrative mismanagement, and the loss of specialty services further increase the risks of closure. Community characteristics such as lower market share, high population density, and proximity to other hospitals, particularly in areas with higher Black and Hispanic populations, also influence hospital closures. These existing studies collectively identified financial distress, market competition, workforce shortages, policy limitations, and community demographics as key drivers of rural hospital closures.

### Limitations identified in the included studies on rural hospital closures

The research on rural hospital closures and their financial viability has several notable limitations. One significant issue is measurement errors, particularly in hospital profitability data derived from unaudited Medicare cost reports and administrative datasets, which may introduce inaccuracies. Additionally, potential reporting errors in these datasets further complicate financial assessments. Many studies define rural hospital markets using commuting zones or county-level data; however, these approaches may not fully capture competitive boundaries or localized economic effects. Another concern is the generalizability of the findings, as results are often limited to specific states or time periods, making them less applicable to hospitals operating under different market conditions or policy environments.

Furthermore, the observational design of many studies hinders the establishment of causal relationships, such as those between Medicare Advantage penetration and financial outcomes or between telehealth adoption and hospital performance. Certain analyses were constrained by small sample sizes, particularly in studies on labor and delivery unit closures, where missing data on maternal care levels and patient demographics limited the scope of the findings. Workforce projections are also subject to uncertainty due to assumptions about population growth and specialization trends. Additionally, the exclusion of certain surgical specialties may have led to an underestimation of workforce shortages.

Studies examining hospital affiliation and closure risk often struggle to differentiate between formal and informal system memberships, and some fail to consider ownership type as a contributing factor. Many studies relied on retrospective designs, which may introduce recall bias into participants’ accounts of hospital closures. Furthermore, the reliance on self-reported survey responses raises concerns about reporting bias. While stepwise regression methods help isolate predictors, they may also limit the generalizability of findings. Several studies also did not fully account for policy changes, such as Medicaid expansion, which could significantly impact hospital sustainability. Additionally, some studies excluded government-run rural hospitals because of their unique financing mechanisms, potentially leading to an overestimation of financial viability among the hospitals studied.

Finally, the inability to observe real-time decision-making processes among hospital executives, along with fiscal year misalignments with calendar years, may have compromised data accuracy in studies assessing financial distress and closure determinants. These limitations underscore the complexity of evaluating rural hospital viability and suggest that future research should integrate more comprehensive data sources, employ refined methodological approaches, and consider broader healthcare outcomes.

## Discussion

The closure of rural hospitals is a critical issue that significantly affects healthcare access and economic stability in communities across the U.S. Numerous studies have identified financial distress, workforce shortages, policy and economic factors, and market dynamics as primary factors contributing to these closures. While previous research has examined these factors individually, this synthesis provides a more integrated perspective, illustrating their interconnected effects on hospital sustainability.

A key limitation identified in prior studies is their predominant reliance on traditional statistical methods to analyze rural hospital closures. These studies often suffer from several limitations, including small sample sizes, limited generalizability beyond specific regions or states, reliance on county-level data, exclusion of key variables such as socioeconomic and health outcomes, lack of causal inference due to observational study designs, and unmeasured confounding factors that impact hospital system affiliations and financial viability. To address these shortcomings, future research should incorporate ML models and multimodal data analysis to more effectively identify the underlying causes of rural hospital closures. Understanding these causes is a crucial step toward developing a real-time forecasting model that can predict and signal potential closures, thereby providing administrators and policymakers with early warning of the risk of closures, allowing them to take timely action to prevent them and avoid the negative impact on communities.

Although Park et al. [21] successfully used AI models to predict clinic closures in Korea using administrative health data, their study focused on outpatient clinics. It did not account for the unique structural, policy, and demographic challenges of rural hospitals. Our review further highlights the absence of real-time predictive frameworks tailored to the U.S. rural hospital context, underscoring a significant opportunity to develop AI-based models for early warning systems. Researchers can build on the insights, predictors, and data sources identified in this review to develop ML models that detect early signs of distress and prevent future closures in rural hospital settings.

Financial distress has consistently been the leading cause of rural hospital closures. Studies highlight declining profitability, low occupancy rates, and heavy reliance on Medicare and Medicaid reimbursements as significant stressors. Hospitals with lower profit margins, high debt levels, and inadequate liquidity are at substantially higher risk of closure. These findings align with research by Bai et al. and Holmes et al., which emphazise that financial viability is crucial for the survival of rural hospitals [20, 33].

A crucial insight from existing research is that Medicaid expansion mitigates hospital closures. Studies by Lindrooth et al. indicate that states that expanded Medicaid experienced lower closure rates, likely due to increased insurance coverage and reduced uncompensated care [41]. However, while Medicaid expansion has helped stabilize some hospitals, its effectiveness varies, particularly in regions with high uninsured rates. Additionally, recent legislative proposals (in 2025) that would significantly reduce federal Medicaid funding could negatively impact states that previously expanded Medicaid under the Affordable Care Act. Such cuts could threaten health care access for millions, particularly in rural areas where hospitals heavily depend on Medicaid reimbursements, underscoring that Medicaid expansion alone is not a comprehensive solution, as shifting federal policies could further compromise rural hospitals’ financial stability.

Rural hospitals also face severe workforce shortages, especially in specialized fields such as obstetrics, emergency medicine, and surgical care [45, 46]. The difficulty in recruiting and retaining healthcare professionals has led to service reductions, jeopardizing hospital sustainability [47, 48]. The shortage of surgeons, obstetricians, and other specialists has been a growing crisis, contributing to hospital closures. Research by Ellison et al. and Hung et al. highlights the declining availability of labor and delivery units and surgical services, primarily due to low patient volumes and financial constraints, particularly in marginalized communities [37, 39]. These closures disproportionately impact pregnant women in rural areas, exacerbating health equity concerns.

Beyond hospital closures, workforce shortages force residents in affected regions to travel longer distances for essential healthcare, potentially leading to poorer health outcomes. Policy interventions, such as loan-forgiveness programs for rural healthcare providers and incentives for residency placements in rural hospitals, could help address these workforce gaps and support the long-term viability of rural healthcare systems.

Market competition presents additional challenges for rural hospitals as they struggle to compete with larger healthcare networks and urban facilities. While mergers and acquisitions have provided financial stability for some hospitals, they often result in reduced access to care, service limitations, or the closure of less profitable departments. Although telemedicine has been proposed as a solution to address service gaps, challenges remain regarding sustainability, reimbursement structures, and the digital divide within rural communities, particularly the lack of access to high-speed broadband and the lack of familiarity with digital tools, which together limit the feasibility of virtual care delivery [49, 50].

Studies indicate that hospitals operating independently, without affiliation with a larger health system, are at higher risk of closure due to financial instability [44]. Independent hospitals typically lack the bargaining power, shared financial resources, and operational efficiencies that make them more resilient in the face of financial distress. While affiliations with larger systems can provide financial support, they do not always prevent closures. Research by Carroll et al. reveals that 7% of unprofitable hospitals closed, and 17% merged with larger systems [35]. However, these mergers did not always lead to improved sustainability; in some cases, parent organizations eliminated unprofitable services to enhance financial performance, inadvertently reducing healthcare access in rural areas. These findings raise an important policy question: Should rural hospitals be encouraged to affiliate with larger networks, or should policymakers focus on sustaining independent hospitals through targeted subsidies and financial incentives?.

Telehealth has been examined as both a potential solution and a financial risk for rural hospitals. Some studies suggest that telemedicine improves healthcare access by reducing the need for costly in-person visits [51]. However, its financial impact on rural hospitals’ sustainability remains uncertain. Cornaggia et al. argue that telehealth could divert revenue from rural hospitals to larger urban facilities, thereby increasing the risk of closure [42], occurs because urban hospitals and health systems can expand their patient base by offering virtual services to individuals in rural areas, effectively drawing patients (and their associated revenue) away from local rural providers, which may already be operating with limited margins.

Recent Medicare and Medicaid telehealth policy updates have extended pandemic-era flexibilities until March 31, 2025. These updates allow rural patients to receive telehealth services from home, expand provider eligibility, and permit audio-only services for mental health and behavioral healthcare. While these changes provide temporary relief, long-term reimbursement stability remains uncertain. Policymakers must carefully design telehealth reimbursement models to prevent financial losses for rural hospitals. Additionally, Medicare and Medicaid policies should be adapted to ensure that rural hospitals benefit from telehealth expansion rather than face revenue reductions. Furthermore, findings by Thomas et al. suggest that rural hospitals in areas with a higher proportion of Black residents face an elevated risk of closure, emphasizing the role of sociodemographic factors in hospital sustainability [36].

Developing real-time AI/ML-based early warning systems will depend on effectively translating existing administrative datasets into dynamic, high-granularity features. For instance, CMS Cost Reports and POS files can provide rolling financial indicators such as quarterly operating margins, liquidity ratios, and reimbursement delays. AHA Annual Surveys and AMA Master Files offer insights into trends in service utilization, market competition, and facility closures. Similarly, HCUP SID and SCHSR datasets can inform service utilization, market competition, and closure trends. At the same time, ACS and BLS data can provide socioeconomic and employment variables that capture community-level vulnerability. When integrated into a continuously updating architecture, these variables can serve as multidimensional input features for AI/ML models that can learn temporal dependencies and generate dynamic risk forecasts. This granular, multi-source integration moves predictive research beyond static financial modeling toward a continuously adaptive surveillance system capable of detecting hospital distress trajectories in near real time.

Despite the promise of AI-driven early warning systems, their impact ultimately depends on whether decision-makers act on the insights they generate [52, 53]. Evidence from other domains, such as epidemic forecasting, climate modeling, and financial risk prediction, shows that even accurate predictive tools are often underutilized when users lack trust, interpretability, or the operational capacity to respond effectively [54, 55]. Rural hospital administrators and policymakers face similar barriers, including competing priorities, limited analytical infrastructure, and political pressures that may lead to delayed or inconsistent responses to risk signals [4]. Therefore, the design of any AI-enabled early warning system must go beyond predictive accuracy to incorporate human-centered design principles that enhance usability, explainability, and decision relevance.

To support this transition, we aim to introduce the Rural Health Control Tower (RHCT), a cloud-based AI-assisted decision-support framework designed to continuously monitor hospital- and community-level indicators, visualize evolving risk trajectories, and issue tiered alerts before distress becomes irreversible. Conceptually, the RHCT seeks to bridge the gap between prediction and action by integrating early detection, decision-support guidance, and transparent communication pathways, representing a shift from reactive crisis response toward proactive, data-driven rural health system management. In this sense, the challenge is not only to build systems that detect risk early but also to foster decision environments in which those warnings are trusted, understood, and acted upon promptly to prevent closure [56].

Unlike traditional retrospective models that identify distress only after deterioration becomes critical, the RHCT would provide an adaptive, forward-looking dashboard that issues tiered alerts to administrators, regulators, and community stakeholders. These alerts could range from short-term operational concerns (e.g., declining liquidity or staffing imbalances) to long-term structural risks (e.g., sustained margin erosion or policy-driven reimbursement changes). By translating complex data into actionable insights, the RHCT aims to shift hospital management and policymaking from a reactive to a proactive stance, enabling early mobilization of financial or workforce interventions before closure becomes inevitable.

The value of real-time analytics lies in its ability to shorten the lead time between risk detection and corrective action. While retrospective FDI approaches, such as those by Holmes et al., are helpful for post hoc analysis [20], they offer limited capacity for preemptive mitigation. In contrast, real-time AI monitoring could “buy time” for hospital leaders and policymakers, enabling decisions such as debt restructuring, service line optimization, or emergency funding applications months before liquidity crises manifest. For regulators and investors, real-time alerts could support dynamic resource allocation, while communities could use these insights to plan for service continuity or alternative care arrangements. This transition from descriptive analytics to prescriptive intervention represents a fundamental paradigm shift in how rural health sustainability is approached.

Stakeholder-level benefits of AI integration are multifaceted. For hospital management teams, AI tools can guide capital allocation, optimize staffing, and forecast operational bottlenecks. For boards and owners, predictive intelligence supports early strategic decision-making and investment realignment. For regulators, real-time surveillance of hospital health allows for timely policy responses. At the same time, for communities, early alerts can help preserve access through coordinated conversion to Rural Emergency Hospital models or regional partnerships. However, realizing these benefits requires addressing several barriers, including data fragmentation, uneven digital infrastructure, and limited analytic capacity in rural systems. Moreover, potential risks such as algorithmic bias, data privacy violations, and overreliance on automation must be carefully managed through transparent governance, ethical AI design, and continuous human oversight.

As AI evolves toward agentic, context-aware architectures, future iterations of early warning systems could extend beyond passive prediction to include autonomous reasoning and decision support. Independent AI platforms capable of contextual planning and simulation could prioritize intervention pathways or automatically recommend policy actions tailored to specific rural contexts. For example, such systems could simulate the effects of telehealth reimbursement policy changes or workforce subsidy programs, offering policymakers a data-informed view of potential outcomes before implementation. Significantly, while these systems may enhance efficiency and reduce manual workloads, they must be developed to complement, not replace, human judgment, empathy, and accountability in healthcare decision-making.

### Research gaps and future directions

Future research should focus on several key areas to enhance our understanding of rural hospital closures. First, there is a critical need to move beyond descriptive and cross-sectional studies toward longitudinal, integrative research that tracks financial performance, workforce stability, and policy environments over time. Such studies should not only document closure events but also model their precursors using adaptive, time-series data to capture early indicators of distress. In addition, there is a pressing need to move beyond traditional statistical approaches toward AI/ML-based analyses capable of identifying and modeling the complex, interdependent drivers of rural hospital closures. Unlike many current models, which often rely on limited financial or utilization metrics that are updated infrequently, AI/ML methods can integrate diverse, real-time inputs, including financial data, workforce trends, policy shifts, and community-level socioeconomic indicators, to detect subtle patterns of risk that might otherwise go unnoticed. By combining these inputs across multiple time horizons, AI/ML systems can identify evolving risk trajectories rather than static thresholds, thereby enhancing both prediction accuracy and policy responsiveness. While ensuring transparency in decision-making, minimizing algorithmic bias, and enhancing generalizability across diverse rural settings remain essential, the potential to deliver timely, actionable insights makes AI/ML the logical next step in advancing early warning systems for rural hospitals.

While this review identifies AI/ML as a promising next step for predicting rural hospital closures, it is important to acknowledge and build on the contributions of existing statistical models, such as those developed by Holmes et al. [20] and Malone et al. [19]. These models, which often rely on logistic regression or other traditional statistical techniques, have successfully identified financial distress indicators and market variables associated with closure risk. However, they are typically limited by static datasets, infrequent updates, and narrower variable sets, which constrain their predictive lead time. Future research should therefore empirically compare the predictive performance, interpretability, and lead time of AI/ML-based systems against these established benchmarks, ensuring that proposed innovations offer demonstrable improvements in real-world forecasting accuracy and decision support. By contrast, AI/ML methods can integrate streaming financial, workforce, and policy data, adapt dynamically to emerging trends, and detect non-linear interactions between variables that traditional methods may overlook. Evaluating the scalability and cross-context generalizability of such models across different rural hospital environments will also be essential for policy translation and system-wide implementation.

Although none of the studies in this review used ML techniques, future research could greatly benefit from AI/ML applications. While developing and validating specific predictive models is beyond the scope of this review, future studies should prioritize the design, piloting, and validation of prototype early warning systems that can be tested across multiple rural contexts to assess their accuracy, user trust, and implementation feasibility. Advanced analytical approaches such as time-series forecasting, survival analysis, and ensemble learning could improve both the accuracy and timeliness of closure risk detection. By leveraging a broad and dynamic dataset that reflects the full spectrum of factors identified in this review, early warning systems could be transformed from static, retrospective tools into proactive decision-support resources. Organizations and community leaders affected by past closures have often noted that these events occur with little warning, leaving minimal time to prepare or implement mitigation strategies [57]. An empirically validated AI/ML-based early warning framework could address this gap by issuing tiered risk alerts, short-term, medium-term, and long-term, enabling policymakers and administrators to mobilize financial, operational, and workforce resources in advance of impending distress.

While financial distress, workforce shortages, and policy challenges are well established as major contributors to rural hospital closures, their interactions remain understudied. Early detection through an AI/ML system could enable targeted responses before these factors reach a crisis point, with strategies differentiated by whether the driver is controllable or uncontrollable. For controllable factors such as deteriorating financial performance, hospitals could renegotiate debt terms, diversify service lines, secure bridge funding, or initiate strategic partnerships before liquidity becomes critical. For workforce shortages, a partially controllable factor, administrators could accelerate recruitment incentives, cross-train existing staff, or expand telehealth capacity to reduce reliance on on-site personnel. For largely uncontrollable drivers such as abrupt state or federal policy changes, early warning could still give leadership time to advocate for regulatory waivers, adjust billing practices, or join coalitions to influence pending legislation. In the face of market pressures, early recognition could distinguish between controllable elements, such as service mix or marketing strategy, and less controllable ones, such as demographic shifts; both could inform decisions about whether to reconfigure services, pursue targeted outreach, or collaborate with nearby providers to share resources and reduce duplication. Future research should explicitly model these interdependencies using systems-based and causal inference frameworks, allowing for simulation of how shifts in one domain (e.g., workforce attrition) propagate through financial and policy systems to increase closure risk. This multilevel, dynamic approach would better capture the complex feedback loops characteristic of rural health ecosystems.

A more comprehensive approach to hospital sustainability research should also incorporate broader economic indicators, such as household debt levels, regional payer mix shifts, and labor market fluctuations, alongside hospital-level factors such as management quality, workforce specialization, and governance structures, to improve both risk assessments and the relevance of early warning systems. Future research should examine how macroeconomic shocks and regional labor trends interact with hospital-level vulnerabilities to predict hospital closure risk across economic cycles. Policy-oriented studies should further evaluate the long-term effects of Medicaid expansion, telehealth reimbursement models, and hospital consolidation regulations on rural healthcare access and financial stability. Given the growing role of telemedicine, it is critical to assess how reimbursement structures affect rural hospitals’ revenue streams and whether telehealth expansion improves sustainability or creates unintended financial risks. Ultimately, by integrating these diverse lines of inquiry: data integration, equity-focused model design, longitudinal policy evaluation, workforce analytics, and real-world validation, future research can advance from documenting closure risks to developing evidence-based, AI-informed interventions that proactively safeguard rural healthcare infrastructure. By combining these insights with predictive modeling, stakeholders could transition from reacting to closures to proactively preventing them, ensuring that rural communities retain access to essential healthcare services and that closures are prevented rather than merely documented after they occur.

In summary, this review highlights five key future research directions:

- Develop longitudinal, multilevel models that integrate financial, policy, and workforce data to improve temporal prediction of closure risks.
- AI/ML-driven frameworks must be designed to enhance predictive accuracy while ensuring transparency, fairness, and contextual equity across diverse rural populations.
- Empirical comparisons between AI-based systems and traditional statistical models should be conducted to establish the added value of advanced analytics.
- Workforce-related metrics should be incorporated into predictive frameworks to understand how labor dynamics interact with financial distress.
- Pilot implementation and validation studies of AI-driven early warning systems such as RHCT are needed to translate predictive insights into actionable, community-informed interventions.

Together, these directions form a coherent, forward-looking research agenda to strengthen rural hospital sustainability and inform proactive, data-driven policy responses.

### Limitations of this review

This review has several limitations. First, the search was restricted to studies published between 2013 and 2024, potentially excluding relevant research on rural hospital closures from earlier years. Second, since this review relies on published literature, the findings are limited to existing studies and may not capture unpublished data or real-time trends in rural hospital closures. Additionally, the studies included in this review vary in their methodologies, datasets, and analytical frameworks, which might lead to inconsistencies in the reported findings. Finally, this review primarily focuses on U.S. rural hospitals, which may limit the generalizability of the findings to rural healthcare systems in other countries.

## Conclusion

Rural hospital closures remain an urgent issue that threatens access to care and community stability. This systematic review identified five key domains: financial distress, workforce shortages, policy influences, economic and demographic pressures, and market competition, which collectively drive instability in rural hospitals. Despite the research examining these factors, no studies to date have applied AI/ML to predict or prevent closures. The evidence highlights an urgent need to develop dynamic, data-driven early warning systems capable of integrating multi-source, real-time information to identify hospitals at risk before closure becomes noticeable. The findings suggest that developing predictive frameworks using AI/ML could transform hospital closure monitoring from a reactive to a proactive process. By integrating diverse data sources, including CMS Cost Reports, AHA Annual Surveys, AMA Master Files, and socioeconomic datasets from the ACS and BLS, future models can generate time-sensitive insights into financial, operational, and workforce trajectories. This multidimensional approach would enable health systems, policymakers, and communities to intervene early with targeted support strategies, such as workforce stabilization, reimbursement adjustments, or capital assistance programs.

However, the success of such systems will depend not only on predictive accuracy but also on transparency, equity, and usability. Developing interpretable models that rural hospital administrators and policymakers can trust will be essential to ensure adoption and impact. Human-centered design principles, emphasizing explainability, ethical governance, and stakeholder engagement, must guide future AI applications to prevent algorithmic bias and promote equitable decision-making across diverse rural settings. In summary, this review highlights the need for a paradigm shift in how rural hospital sustainability is studied and supported. By advancing from retrospective statistical models to adaptive, AI-driven early warning frameworks, such as the proposed RHCT concept, researchers and policymakers can move toward real-time surveillance, timely intervention, and sustainable rural healthcare delivery. Building these systems will require collaborative investment across healthcare, policy, and technology sectors, but the potential benefits, preserved access, economic stability, and healthier rural communities, make this a necessary and achievable goal for the future of rural health in the U.S.

## Electronic supplementary material

Below is the link to the electronic supplementary material.

Supplementary Material 1

Supplementary Material 1

## Data Availability

All data generated or analyzed during this study are included in this published article.
