# Supplementary material for: Predictors of rural hospital closures in the United States: a systematic review and call for AI-driven early warning systems
Source: BMC Health Serv Res. 2025 Dec 13;26:86. doi: 10.1186/s12913-025-13847-7 (PMC12822351; doi:10.1186/s12913-025-13847-7)
Supplement: Supplementary file 1 — Supplementary Material 1 [file 12913_2025_13847_MOESM1_ESM.docx]

**TABLE S1: Summary of studies included in narrative synthesis**

| **Author, Year, Title** | **Objective** | **Methodology** | **Data used** | **Reason for Rural Hospital Closure** |
| --- | --- | --- | --- | --- |
| Smith, et al., 2022, It's not right: Nurse perspectives on rural hospital closures: A qualitative study[22] | To describe rural hospital closure contributors, processes, and outcomes from the perspective of licensed rural nurses in a hospital during its closure. | Cross-sectional retrospective qualitative study using thematic analysis of semi-structured interviews with 10 rural nurses. | Semi-structured interviews were conducted with 10 nurses from two rural Texas hospitals between 2014 and 2015. | Financial instability, policy changes, lack of community support, administrative mismanagement, and loss of specialty services. |
| Bai, et al., 2020, Varying Trends In The Financial Viability Of U.S. Rural Hospitals, 2011–17 [32]. | To analyze changes in financial viability across various rural hospital types and determine factors influencing this viability. | Longitudinal analysis, descriptive, and regression analyses. | Centers for Medicare & Medicaid Services (CMS) hospital cost reports and Small Area Health Insurance Estimates from the U.S. Census Bureau (U.S. CB). | Declining profit margins, low occupancy rates, lack of Medicaid expansion, and high dependency on Medicare/Medicaid payments. |
| Holmes, et al. 2016, Predicting Financial Distress and Closure in Rural Hospitals [20]. | To develop and validate a Financial Distress Index (FDI) to predict financial distress and closures in rural hospitals within two years. | Logistic regression model using financial, organizational, and market characteristics to predict distress; validated using a c-statistic. | CMS Healthcare Cost Report Information System (HCRIS, “Medicare Cost Reports”) and Provider of Services (POS) ﬁles for ﬁnancial variables and Nielsen-Claritas Population Facts for the market variables. | Low profitability, smaller size, for-profit status, high competition, poorer community economic conditions, low government reimbursement, and lack of Critical Access Hospital designation. |
| Henke, et al., 2023, Medicare Advantage in Rural Areas: Implications for Hospital Sustainability [33]. | Examine the impact of Medicare Advantage (MA) penetration levels on rural hospital financial distress and closure. | A retrospective cohort study (2008-2019) used fixed-effects regression and Cox proportional hazard models. | Healthcare Cost and Utilization Project (HCUP) State Inpatient Databases(SID), CMS Medicare Advantage enrollment data, SCHSR, AHA Annual Survey, CMS Cost Reports, and American Community Survey (ACS). | Financial distress (low Altman Z-score), high competition, low reimbursement rates, and small market size. |
| Carroll, et al., 2023, Hospital Survival in Rural Markets: Closures, Mergers, and Profitability [34]. | To examine the impact of financial distress on rural hospital survival, including closures and mergers, and implications for access to care and market competition. | Multinomial logit analysis and descriptive statistics to evaluate closures, within-market mergers, and out-of-market mergers among rural hospitals between 2010–2018. | CMS Hospital Cost Report Information System, SCHSR, American Hospital Directory, National Bureau of Economic Research, Irving Levin Associates (ILA). | Declining patient volumes, financial unprofitability, Medicare's lower reimbursements compared to private payers, and competition in rural areas. |
| Thomas, et al., 2016, To What Extent do Community Characteristics Explain Differences in Closure among Financially Distressed Rural Hospitals? [35]. | To examine how community characteristics contribute to rural hospital closures among financially distressed hospitals from 2005-2015. | Comparative analysis of closed and financially distressed but open rural hospitals using multilevel logistic regression. | CMS Hospital Cost Report, POS, Nielsen-Claritas Pop-Facts data, and Rural-Urban Commuting Area (RUCA), Hospital service area file | Lower market share, high population density, proximity to other hospitals, higher unemployment rates, and a higher percentage of Black and Hispanic populations. |
| Hung, et al. 2016, Why Are Obstetric Units in Rural Hospitals Closing Their Doors? [36]. | To identify hospital- and county-level factors influencing rural obstetric unit closures and understand post-closure prenatal care access. | Mixed-methods approach; multivariate logistic regression and qualitative analysis. | HCUP SID, AHA Annual Survey, Area Resource File (ARF), and telephone interviews of 306 rural hospitals across 9 states. | Low birth volume, difficulty in staffing (obstetricians, surgical, and anesthesia coverage), financial issues including low reimbursement rates, budget cuts, and high Medicaid patient population. |
| Daymude, et al., 2022, Labor and Delivery Unit Closures in Rural Georgia from 2012 to 2016 and the Impact on Black Women [37]. | To explore factors associated with labor and delivery unit (LDU) closures in rural Georgia from 2012 to 2016, focusing on the disproportionate impact on Black women. | Mixed-methods study: Quantitative analysis of 2011 regional, hospital, and patient data; qualitative analysis of 18 newspaper articles. | Georgia Department of Public Health databases, Online Analytical Statistical Information System (OASIS), U.S. CB, Georgia Board of Health Care Workforce, Georgia Maternal and Infant Health Research Group (GMIHRG), Emory's MCH Linked Vital Records Data Repository. | Financial losses from obstetric services, low birth volumes, inadequate Medicaid reimbursements, high uninsured/self-pay rates, and shortages of obstetric providers. |
| Ellison et al., 2021, The Continued Urbanization of American Surgery: A Threat to Rural Hospitals [38]. | To evaluate rural hospitals and surgeon shortages, update population and workforce trends, and project the demand for rural and urban surgeons by 2040. | Quantitative analysis of population trends, surgeon supply and demand, and rural vs. urban workforce needs. | U.S. CB, American Board of Medical Specialties certifications, American Medical Association (AMA), and American Hospital Association (AHA) hospital statistics. | Surgeon shortages, inability to recruit due to financial disadvantages, low surgical volume, dependence on Medicaid/Medicare reimbursement, and the continued urbanization of surgical services. |
| Lindrooth et al., 2018, Understanding The Relationship Between Medicaid Expansions And Hospital Closures [39]. | To examine how Medicaid expansion under the Affordable Care Act (ACA) impacts hospital financial stability and closures, particularly in rural areas. | Difference-in-differences analysis and logistic regression using hospital financial and closure data from 2008-2016. | CMS, U.S. CB, KFF, State-level Medicaid and Children's Health Insurance Program (CHIP), CMS Healthcare Provider Cost Reporting System, Herfindahl-Hirschman Index (HHI). | Higher rates of uninsured patients leading to financial strain, lack of Medicaid expansion increasing uncompensated care, rural hospital vulnerabilities due to low patient volumes |
| Cornaggia et al., 2024, Financial Effects of Remote Product Delivery: Evidence from Hospitals [40]. | To study the financial effects of telehealth and its impact on rural hospital operations and closures. | Quantitative study using financial data and staggered adoption of telehealth parity laws as a quasi-natural experiment. | National Center for Health Statistics (NCHS), Municipal Bond Ratings History, IBM Marketscan, CMS, Mergent Municipal Fixed Income Database, Municipal Securities Rulemaking Board (MSRB), Bureau of Economic Analysis (BEA), Bureau of Labor Statistics (BLS), and Centers for Disease Control and Prevention (CDC). | Redistribution of revenue from rural to urban hospitals due to telehealth leading to reduced revenue, profit margins, credit rating downgrades, and higher financial distress for rural hospitals. |
| Rhoades et al., 2022, Community Sociodemographics and Rural Hospital Survival [41]. | To determine whether community sociodemographic factors are associated with the survival or closure of rural hospitals at risk of financial distress between 2010 and 2019. | Wilcoxon rank-sum tests to identify community sociodemographic differences and multilevel Weibull proportional hazards regression for survival analysis. | AHA Annual Survey, SCHSR US CB, ACS, Robert Wood Johnson Foundation, County Health Rankings & Roadmaps, BLS | Higher community unemployment rates, higher uninsured populations under 65, and increased uncompensated care burdens. |
| Chatterjee, et al., 2022, Changes in Economic Outcomes Before and After Rural Hospital Closures in the United States: A Difference-in-Differences Study [42]. | To assess changes in local economic outcomes before and after rural hospital closures. | Difference-in-differences study using county-level and commuting-zone-level data. | SCHSR, economic data from the Bureau of Labor Statistics,Bureau of Economic Analysis, Quarterly Workforce Indicators, U.S. Federal Reserve Economic Data, RAND Corporation state statistics database, U.S. Social Security Administration, and U.S. CB. | Adverse economic conditions preceding closures (unemployment, income declines). |
| Kaufman et al., 2016, The Rising Rate of Rural Hospital Closures [43]. | To analyze the factors leading to rural hospital closures in the U.S. and identify financial and market-related risks. | Comparative financial and market characteristics analysis using Pearson’s chi-square and Wilcoxon rank tests. Logistic regression for predicting financial distress. | U.S. Department of Health and Human Services, CMS, Provider of Services file (POS), Provider-Specific File (PSF), Nielsen-Claritas Pop Facts database, Hospital Service Area File | Low profitability, low liquidity, high debt, small market share, small population, high reliance on Medicare and Medicaid, competition from larger hospitals |
| Jiang, et al., 2022, Risk of Closure Among Independent and Multihospital-Affiliated Rural Hospitals [44]. | To examine the association between affiliation and rural hospital closure. | Cohort study using survival models with a time-dependent variable for affiliation vs independent status. | AHA Annual Survey, ILA, SCHSR, HCUP SID, CMS Cost Reports ACS, HCUP, Kaiser Family Foundation (KFF). | Financial distress, affiliation with multihospital systems, proprietary ownership, and reduced market share. |
