# Supplementary material for: Predictors of rural hospital closures in the United States: a systematic review and call for AI-driven early warning systems
Source: BMC Health Serv Res. 2025 Dec 13;26:86. doi: 10.1186/s12913-025-13847-7 (PMC12822351; doi:10.1186/s12913-025-13847-7)
Supplement: Supplementary file 2 — Supplementary Material 1 [file 12913_2025_13847_MOESM2_ESM.docx]

Pubmed:
((("hospitals, rural"[MeSH Terms] OR ("hospitals"[All Fields] AND "rural"[All Fields]) OR "rural hospitals"[All Fields] OR ("hospital"[All Fields] AND "rural"[All Fields]) OR "hospital rural"[All Fields]) AND ("closure"[All Fields] OR "closure s"[All Fields] OR "closures"[All Fields])) OR ("health facility closure"[MeSH Terms] OR ("health"[All Fields] AND "facility"[All Fields] AND "closure"[All Fields]) OR "health facility closure"[All Fields])) AND ((("Rural Health Services"[MeSH Terms] OR "rural population"[MeSH Terms] OR "hospitals, rural"[MeSH Terms] OR "rural area"[Title/Abstract] OR "rural areas"[Title/Abstract]) AND ("health services administration"[MeSH Terms] OR "insurance, health, reimbursement"[MeSH Terms] OR "reimbursement, disproportionate share"[MeSH Terms] OR "cost"[Title/Abstract] OR "costs"[Title/Abstract] OR "costs and cost analysis"[MeSH Terms] OR "economics"[MeSH Subheading] OR "qualitative"[Title/Abstract] OR ("socioeconomic factors"[MeSH Terms] OR ("socioeconomic"[All Fields] AND "factors"[All Fields]) OR "socioeconomic factors"[All Fields] OR "socioeconomics"[All Fields] OR "socioeconomic"[All Fields] OR "socioeconomical"[All Fields] OR "socioeconomically"[All Fields]) OR "cost effectiveness*"[Title/Abstract] OR (("workforce"[MeSH Terms] OR "workforce"[All Fields] OR "workforces"[All Fields] OR "workforce s"[All Fields]) AND ("shortage"[All Fields] OR "shortages"[All Fields])) OR (("infrastructural"[All Fields] OR "infrastructure"[All Fields] OR "infrastructures"[All Fields]) AND ("limit"[All Fields] OR "limitation"[All Fields] OR "limitations"[All Fields] OR "limited"[All Fields] OR "limiting"[All Fields] OR "limits"[All Fields])) OR ("low"[All Fields] AND ("patient s"[All Fields] OR "patients"[MeSH Terms] OR "patients"[All Fields] OR "patient"[All Fields] OR "patients s"[All Fields]) AND ("volum"[All Fields] OR "volume"[All Fields] OR "volumes"[All Fields] OR "voluming"[All Fields])) OR "Health services research"[MeSH Terms] OR "health care quality, access, and evaluation"[MeSH Terms] OR "Health Status Disparities"[MeSH Terms] OR "socioeconomic factors"[MeSH Terms] OR "healthcare disparities"[MeSH Terms] OR "Health services research"[Title/Abstract] OR "PHSSR"[Title/Abstract] OR ("public health"[All Fields] AND "services"[Title/Abstract] AND "systems"[Title/Abstract])) AND "english"[Language]) NOT ("south America"[MeSH Terms] OR "asia"[MeSH Terms] OR "europe"[MeSH Terms] OR "africa"[MeSH Terms] OR "canada"[MeSH Terms] OR "mexico"[MeSH Terms] OR "australia"[MeSH Terms]))

Embase:
('health facilities closures' OR (('health'/exp OR health) AND facilities AND closures) OR (('rural hospital'/exp OR 'rural hospital') AND closures)) AND ('health service'/exp OR 'reimbursement'/exp OR 'cost':ti,ab,kw OR 'costs':ti,ab,kw OR 'cost'/exp OR 'economics' OR 'qualitative':ti,ab,kw OR ('socioeconomic' AND 'factors') OR 'socioeconomic factors' OR 'socioeconomics' OR 'socioeconomic' OR 'socioeconomical' OR 'socioeconomically' OR 'cost effectiveness*':ti,ab,kw OR (('workforce'/exp OR 'workforce' OR 'workforces' OR 'workforce s') AND ('shortage' OR 'shortages')) OR (('infrastructural' OR 'infrastructure' OR 'infrastructures') AND ('limit' OR 'limitation' OR 'limitations' OR 'limited' OR 'limiting' OR 'limits')) OR ('low' AND ('patient s' OR 'patient'/exp OR 'patients' OR 'patient' OR 'patients s') AND ('volum' OR 'volume' OR 'volumes' OR 'voluming')) OR 'health services research'/exp OR 'health care quality'/exp OR 'health disparity'/exp OR 'socioeconomics'/exp OR 'health care disparity'/exp OR 'health services research':ti,ab,kw OR 'phssr':ti,ab,kw OR ('public health' AND 'services':ti,ab,kw AND 'systems':ti,ab,kw)) AND ('united states'/exp OR 'united states')

Scopus:
( ( TITLE-ABS-KEY ( health AND facilit* AND closure ) ) OR ( TITLE-ABS-KEY ( rural AND hospital AND closure ) ) ) AND ( TITLE-ABS-KEY ( "Rural Health Services" OR "rural population" OR "rural hospitals" OR "rural area" OR "rural areas" ) ) AND ( ( "health services administration" OR "insurance, health, reimbursement" OR "reimbursement, disproportionate share" OR "cost" OR "costs" OR "costs and cost analysis" OR "economics" OR "qualitative" ) OR ( TITLE-ABS-KEY ( "socioeconomic factors" OR ( "socioeconomic" AND "factors" ) OR "socioeconomic factors" OR "socioeconomics" OR "socioeconomic" OR "socioeconomical" OR "socioeconomically" OR "cost effectiveness*" ) ) OR ( TITLE-ABS-KEY ( ( "workforce" OR "workforce" OR "workforces" OR "workforce s" ) AND ( "shortage" OR "shortages" ) ) ) OR ( TITLE-ABS-KEY ( ( "infrastructural" OR "infrastructure" OR "infrastructures" ) AND ( "limit" OR "limitation" OR "limitations" OR "limited" OR "limiting" OR "limits" ) ) ) OR ( TITLE-ABS-KEY ( "low" AND ( "patient s" OR "patients" OR "patients" OR "patient" OR "patients s" ) AND ( "volum" OR "volume" OR "volumes" OR "voluming" ) ) ) OR ( TITLE-ABS-KEY ( "Health services research" OR "health care quality, access, and evaluation" OR "Health Status Disparities" OR "socioeconomic factors" OR "healthcare disparities" OR "Health services research" OR "PHSSR" OR ( "public health" AND "services" AND "systems" ) ) ) ) AND ( LIMIT-TO ( LANGUAGE , "English" ) ) AND ( LIMIT-TO ( AFFILCOUNTRY , "United States" ) )

CINAHL

(((MH "Health Services Administration") OR "health services administration") OR ((MH "Insurance, Health, Reimbursement")) OR ((MH "Reimbursement, Incentive") OR (MH "Reimbursement Mechanisms")) OR ((MH "Costs and Cost Analysis")) OR (costs OR cost) OR ((MH "Economics")) OR ((MH "Socioeconomic Factors") OR "socioeconomic factors") OR socioeconomic* OR ("cost effectiveness" OR (MH "Cost Effectiveness Analysis")) OR ((MH "Personnel Shortage") OR "workforce shortage") OR (infrastructure limitations) OR ("patient volume") OR ((MH "Health Services Research") OR "health services research") OR ((MH "Quality of Health Care")) OR "health care quality, access, and evaluation" OR ((MH "Healthcare Disparities") OR "healthcare disparities") OR ("public health services" OR (MH "United States Public Health Service")) OR "PHSSR" OR infrastructure) AND ((((MH "Health Facility Closure")) OR ((closures OR closing) AND (((MH "Health Facilities") OR "health facilities") OR ((MH "Hospitals, Rural") OR "rural hospitals")))) AND (((MH "Rural Health Services") OR "rural health services") OR ((MH "Hospitals, Rural") OR "rural hospitals") OR ((MH "Rural Population") OR "rural populations") OR ((MH "Rural Areas") OR "rural area")))
